# Characterization of CMY-2-type beta-lactamase-producing *Escherichia coli* isolated from chicken carcasses and human infection in a city of South Brazil

**DOI:** 10.1186/s12866-019-1550-3

**Published:** 2019-07-30

**Authors:** Vanessa L. Koga, Renato P. Maluta, Wanderley D. da Silveira, Renan A. Ribeiro, Mariangela Hungria, Eliana C. Vespero, Gerson Nakazato, Renata K. T. Kobayashi

**Affiliations:** 1 Basic and Applied Bacteriology Laboratory, Department of Microbiology, State University of Londrina (UEL), Londrina, PR Brazil; 2 Bacterial Molecular Biology Laboratory, Department of Genetics, Evolution and Bioagents, Institute of Biology, State University of Campinas (UNICAMP), Campinas, SP Brazil; 3 Soil Biotechnology Laboratory, Brazilian Agricultural Research Corporation (Embrapa), Londrina, PR Brazil; 4 Department of Pathology and Clinical and Toxicological Analysis, State University of Londrina (UEL), Londrina, PR Brazil

**Keywords:** *Escherichia coli*, Chicken, Plasmid-mediated AmpC, Zoonotic risk

## Abstract

**Background:**

Food-producing animals, mainly poultry, have been associated with the maintenance and dissemination of antibiotic-resistant bacteria, such as plasmid-mediated AmpC (pAmpC)-producing Enterobacteriaceae, to humans, thus impacting food safety. Many studies have shown that *Escherichia coli* strains isolated from poultry and humans infections share identical cephalosporin resistance, suggesting that transmission of resistance from poultry meat to humans may occur. The aim of this study was to characterize pAmpC-producing *E. coli* strains isolated from chicken carcasses and human infection in a restrict area and to determine their antimicrobial resistance profiles, and molecular type by multilocus sequence typing (MLST) and pulsed-field gel electrophoresis (PFGE).

**Results:**

A total of 14 pAmpC-producing *E. coli* strains were isolated, including eight strains from chicken carcasses and six strains from human infections (from urine, tissue and secretion). The *bla*_CMY-2_ gene was identified in all pAmpC-producing *E. coli* strains by polymerase chain reaction (PCR) and DNA sequencing. High percentages of strains resistant to tetracycline, nalidixic acid and sulfamethoxazole-trimethoprim (78–92%) were detected, all of which were considered multidrug-resistant. Among the non-beta-lactam resistance genes, the majority of the strains showed *tet*A, *tet*B, *sul*I and *sul*II. No strain was considered an extended-spectrum beta-lactamases (ESBL) producer, and the *bla*_TEM-1_ gene was found in 2 strains isolated from human infection. Six strains from chicken carcasses and four strains from humans infections were linked to an ISE*cp1*-like element. Through MLST, 11 sequence types were found. Three strains isolated from human infection and one strain isolated from chicken carcasses belonged to the same sequence type (ST354). However, considerable heterogeneity between the strains from chicken carcasses and humans was confirmed by PFGE analysis.

**Conclusion:**

This study showed the prevalence of *E. coli* strains producing *bla*_CMY-2_ linked to ISE*cp1* that were present in both chickens and humans in a restricted area. Our results also suggest the presence of a highly diverse strains that harbor pAmpC, indicating no clonal dissemination. Therefore, continuous monitoring and comparative analyses of resistant bacteria from humans and food-producing animals are needed.

## Background

Food-producing animals have been associated with the maintenance and dissemination of antimicrobial-resistant bacteria to humans, impacting food safety. Studies have indicated that poultry meat is an important reservoir for resistance problems rapidly emerging worldwide due to bacterial selection caused by antimicrobial agents used as growth promoters or for prophylactic and therapeutic purposes [1–4].

In recent years, the frequency of resistance to third-generation cephalosporins has increased both in strains isolated from human infections and from the colonization of food-producing animals, mainly mediated by extended-spectrum beta-lactamases (ESBL) and the AmpC-beta-lactamase. However, the epidemiology of AmpC-producing bacteria may be underreported due to the lack of a phenotypic test for the detection of this mechanism of resistance. Failure to detect this beta-lactamase has contributed to its uncontrolled spread and occasional therapeutic failure [5–8].

Bacteria overexpressing AmpC beta-lactamases are usually resistant to all beta-lactam antibiotics, except cefepime, cefpirome, and carbapenems, which is an important clinical concerns because the bacteria often express a multidrug-resistant phenotype, leaving limited therapeutic options. The AmpC beta-lactamase can be encoded by genes located on chromosomes or plasmids. In *Escherichia coli*, the expression of the chromosome-encoded AmpC beta-lactamase is very low, due to the absence of the *amp*R regulator gene. On the other hand, the genes that encode plasmid-mediated AmpC beta-lactamases (pAmpC) in *E. coli* are often overexpressed and have been found around the world in nosocomial and non-nosocomial isolates. Plasmid-mediated *amp*C genes originated from chromosomal *amp*C genes carried by several gram-negative species and are classified into at least five phylogenetic groups, namely, the *Enterobacter* group (MIR, ACT), the *Citrobacter freundii* group (CMY-2-like, LAT, CFE), the *Morganella morganii* group (DHA), the *Hafnia alvei* group (ACC), and the *Aeromonas* group (CMY-1like, FOX, MOX), with the most prevalent and widely disseminated being CMY-2-like enzymes. The presence of AmpC in plasmids has contributed to the rapid spread of this mechanism of resistance [5, 7, 9].

The prevalence of pAmpC-producing *E. coli* varies significantly depending on the geographical region and host, with a high prevalence in both humans and food-producing animals mainly in North America [2–4]. In Brazil, pAmpC betalactamases were first reported in strains isolated from humans between 2007 and 2008 [10, 11]. Since then, AmpC-producing bacteria have been reported in food-producing animals, such as poultry carcasses [12–16]. However, there are few studies on AmpC-mediated resistance in human clinical and veterinary medicine in Brazil. As Brazil is one of the largest chicken meat exporters in the world and our work has pointed out a significant increase in the presence of beta-lactamases in chicken meat from Brazil [12], an investigation of the spread of AmpC genes in food-producing animals is also important to elucidate the origin of resistant strains. The aim of our study was characterize pAmpC-producing *E. coli* strains from both chicken carcasses and human clinical samples from a city in southern Brazil (Paraná state) within close time periods to determine whether chicken meat might act as a reservoir and dissemination route for pAmpC-producing *E. coli*. These strains were studied regarding their antimicrobial resistance profiles and molecular typing by multilocus sequence typing (MLST) and pulsed-field gel electrophoresis (PFGE).

## Methods

### Bacterial isolates

In 2013, a study performed by our group [12] isolated 121 *E. coli* strains from commercial refrigerated chicken carcasses intended only for local consumption that were sold in a city in southern Brazil. From these strains, 8 were screened and confirmed as pAmpC-producing strains by polymerase chain reaction (PCR) described by Pérez-Pérez and Hanson (2002) [7]. These strains belong to the collection of the Basic and Applied Bacteriology Laboratory from State University of Londrina (UEL), Londrina, PR, Brazil. Between 2013 and 2015, 6 *E. coli* strains isolated from human infection (from urine, tissue and secretion) were confirmed as pAmpC by the Vitek system GNID card (bioMérieux, Marcy I’Etoile, France) and PCR [7] (Table 1). Only one isolate was selected per patient. These strains were provided by the University Hospital of Londrina, Londrina, Paraná, Brazil.

**Table 1:** Oligonucleotide used for amplification in the PCR

Target	Gene	Oligonucleotide sequence (5′ to 3′)	References
*bla*_ESBL_	*bla*_CTX-M-1_ group	F- AAA AAT CAC TGC GCC AGT TC	[17]
		R- AGC TTA TTC ATC GCC ACG TT	
	*bla*_CTX-M2_ group	F- CGA CGC TAC CCC TGC TAT T	
		R- CCA GCG TCA GAT TTT TCA GG	
	*bla*_CTX-M8_ group	F- TCG CGT TAA GCG GAT GAT GC	
		R- AAC CCA CGA TGT GGG TAG C	
	*bla*_CTX-M9_ group	F- CAA AGA GAG TGC AAC GGA TG	
		R- ATT GGA AAG CGT TCA TCA CC	
	*bla*_CTX-M25_ group	F- GCA CGA TGA CAT TCG GG	
		R- AAC CCA CGA TGT GGG TAG C	
	*bla*_TEM_	F- CAT TTC CGT GTC GCC CTT ATT C	[18]
		R- CGT TCA TCC ATA GTT GCC TGA C	
	*bla*_SHV_	F- CAC TCA AGG ATG TAT TGT G	[19]
		R- TTA GCG TTG CCA GTG CTC G	
*bla*_pAmpC_	*bla*_MOX-1_, *bla*_MOX-2_, *bla*_CMY-1_, bla_CMY-8_ to *bla*_CMY-11_	F- GCT GCT CAA GGA GCA CAG GAT	[7]
		R- CAC ATT GAC ATA GGT GTG GTG C	
	*bla*_LAT-1_ to *bla*_LAT-4_, *bla*_CMY-2_ to *bla*_CMY-7_, *bla*_BIL-1_	F- TGG CCA GAA CTG ACA GGC AAA	
		R- TTT CTC CTG AAC GTG GCT GGC	
	*bla*_DHA-1_ and *bla*_DHA-2_	F- AAC TTT CAC AGG TGT GCT GGG T	
		R- CCG TAC GCA TAC TGG CTT TGC	
	*bla*_ACC_	F- AAC AGC CTC AGC AGC CGG TTA	
		R- TTC GCC GCA ATC ATC CCT AGC	
	*bla*_MIR-1T_, *bla*_ACT-1_	F- TCG GTA AAG CCG ATG TTG CGG	
		R- CTT CCA CTG CGG CTG CCA GTT	
	*bla*_FOX-1_ to *bla*_FOX-5b_	F- AAC ATG GGG TAT CAG GGA GAT G	
		R- CAA AGC GCG TAA CCG GAT TGG	
	*bla*_CMY-2_, *bla*_CMY-4_, *bla*_CMY-6_, *bla*_CMY-7_, *bla*_CMY-12_, *bla*_CMY-13_, *bla*_CMY-14_, *bla*_CMY-18_, *bla*_LAT-3_	F- ATG ATG AAA AAA TCG TTA TGC TGC	[20]
		R- GCT TTT CAA GAA TGC GCC AGG	
PMQR	*qnr*A	F- AGA GGA TTT CTC ACG CCA GG	[21]
		R- TGC CAG GCA CAG ATC TTG AC	
	*qnr*B	F- GGM ATH GAA ATT CGC CAC TG	
		R- TTT GCY GYY CGC CAG TCG AA	
	*qnr*S	F- GCA AGT TCA TTG AAC AGG GT	
		R- TCT AAA CCG TCG AGT TCG GCG	
Tetracycline resistance	*tet*A	F- GCC TTT CCT TTG GGT TCT CT	[22]
		R- TGT CCG ACA AGT TGC ATG AT	
	*tet*B	F- GCT TTC AGG GAT CAC AGG AG	
		R- GAC CAA GAC CCG CTA ATG AA	
Sulfonamide resistance	*sul*I	F- ACG AGA TTG TGC GGT TCT TC	[22]
		R- GGT TTC CGA GAT GGT GAT TG	
	*sul*II	F- CCG TCT CGC TCG ACA GTT AT	
		R- GTG TGT GCG GAT GAA GTC AG	
Insertion sequence	ISE*cp1* - CMY	F- AAA AAT GAT TGA AAG GTG GT	[7, 23]
		R- TTT CTC CTG AAC GTG GCT GGC	

### Antimicrobial susceptibility testing

Antimicrobial susceptibility testing of *E. coli* isolates was performed using the standard disk-diffusion method recommended by the Clinical and Laboratory Standards Institute [24, 25], with the following antimicrobials: ciprofloxacin (5 μg), gentamicin (10 μg), norfloxacin (10 μg), enrofloxacin (10 μg), cefotaxime (30 μg), cefoxitin (30 μg), ceftazidime (30 μg), tetracycline (30 μg), nalidixic acid (30 μg), chloramphenicol (30 μg), nitrofurantoin (300 μg), trimethoprim-sulfamethoxazole (1.25/23.75 μg) and amoxicillin-clavulanic acid (20/10 μg) (Oxoid Ltd., Basingstoke, Hants, UK). For the negative control, we used *E. coli* strain ATCC 25922. All strains resistant to 3rd generation cephalosporins were tested for phenotypic confirmation of ESBL production by standard ceftazidime and cefotaxime disks combined with clavulanic acid [25] and by the double-disk diffusion method with disks containing cefepime, cefotaxime, ceftazidime and aztreonam placed 25 mm apart (center to center) to a disk containing a beta-lactamase inhibitor (amoxicillin-clavulanic acid) [26].

### Screening of antimicrobial resistance genes and insertion sequence

All *E. coli* strains were screened by PCR for *bla*_CMY_ gene as described by Dierikx and collaborators (2010) and sequenced [20] (Table 1). For sequencing, amplicons were purified with a column-based kit (Pure Link Quick PCR Purification Kit, Invitrogen, Germany). The purified product was sequenced based on Sanger methodology using an ABI PRISM 3500xL Genetic Analyzer (Applied Biosystems, Foster City, CA). The sequencing was performed at the Multiuser Laboratory of Genotyping and Sequencing from State University of Campinas (UNICAMP) and in the Soil Biotechnology Laboratory from the Brazilian Agricultural Research Corporation (Embrapa).

After sequencing, homology searches were done based on the BLAST algorithm available at http://blast.ncbi.nlm.nih.gov/Blast.cgi. The DNA sequences were compared with reference sequences from the LAHEY home page (http://www.lahey.org/Studies/).

The strains were also analyzed for the presence of other major beta-lactamase genes (*bla*_CTX-M_, *bla*_SHV_, *bla*_TEM_), plasmid mediated quinolone resistance (PMQR) determinants (*qnr*A, *qnr*B and *qnr*S), sulfonamide resistance genes (*sul*I and *sul*II) and tetracycline resistance genes (*tet*A and *tet*B) [7, 17–22]. To define the mechanism of transmission of the pAmpC gene, we screened *bla*_ampC_ for the insertion sequence ISE*cp*1 by ISE*cp*1-*bla*_CMY_ linkage PCR [23]. Strains positive for this PCR were sequenced as previously described. Details regarding oligonucleotide primers and references are illustrated in Table 1. The PCR mixture was prepared using Promega PCR Master Mix (Promega, USA). PCR amplicons were visualized on 2.0% agarose gels stained with GelRed (Biotium). After gel electrophoresis, the images were captured using an Image Capture System (LPixImageHE). Strains positive for beta-lactamases (non AmpC-type) were also sequenced as previously described.

### Multilocus sequence typing

MLST was performed according to the Achtman scheme (http://mlst.warwick.ac.uk/mlst/dbs/Ecoli), for sequencing the PCR amplificon *adk*, *fum*C, *gyr*B, *icd*, *mdh*, *pur*A and *rec*A. Sequencing was performed as previously described in this manuscript.

### Pulsed-field gel electrophoresis

Genomic relationships were analyzed by *Xba*I restriction digestion followed by pulsed-field gel electrophoresis (PFGE) using the CHEF DR III PFGE System (BioRad, Hercules, CA, USA). Electrophoresis conditions consisted of an initial time of 2.2 s, a final time of 54.2 s at a gradient of 6 V cm^− 1^ and an included angle of 120°. The gels were electrophoresed for 18 h. The results were evaluated with BioNumerics (version 7.6; Applied Maths, Austin, TX, USA) using the cut-off value of 80% similarity to distinguish PFGE types.

## Results

**Fig. 1:**
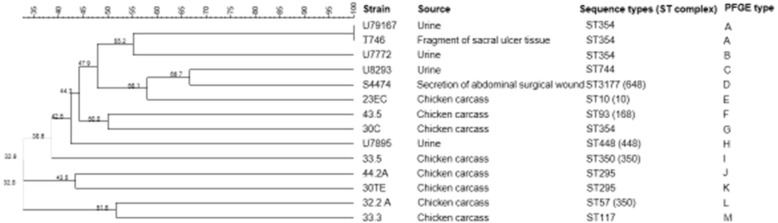
Relationship among pAmpC-producing *E. coli* strains from chicken carcasses and humans infections by PFGE and MLST

A total of 14 strains (8 strains isolated from chicken carcasses and 6 strains isolated from humans infections) were confirmed as pAmpC-producing strains by PCR. The strains from humans infections were isolated from urine (*N* = 4), a fragment of sacral ulcer tissue (*N* = 1) and secretion of an abdominal surgical wound (*N* = 1) (Fig. 1). PCR and sequencing, using specific primers (Table 1) identified the *bla*_CMY-2_ gene in all pAmpC-producing *E. coli* strains.

All the strains were resistant to amoxicillin-clavulanic acid, and 92.86% of the strains were resistant to cefoxitin. According to the antimicrobial susceptibility profile for non-beta-lactam antimicrobials, the strains presented a high frequency of resistance for mainly tetracycline (92.86%), nalidixic acid (92.86%) and sulfamethoxazole-trimethoprim (78.57%) (Table 2). Among the non-beta-lactamase genes, the strains showed *tet*A (7 from chickens and 2 from humans), *tet*B (6 from chickens and 3 from humans), *sul*I (8 from chickens) and *sul*II (7 from chickens and 1 from humans) (Table 2). PMQRs were not found. All strains were considered multidrug-resistant (nonsusceptible to at least 1 agent in 3 or more antimicrobial categories) [27].

**Table 2:** Antimicrobial resistance profiles, presence of resistance genes and ISE*cp1* in AmpC beta-lactamase positive *E. coli* strains

Strains	Antimicrobial resistance profile to non beta-lactams	Betalactamase genes	Non beta-lactam resistance genes	ISE*cp1*
Chicken carcass				
23EC	tet, gen, nal, sut	*bla*_CMY-2_	*tet*A, *tet*B, *sul*I, *sul*II	+
30C	tet, gen, clo, nal, cip, nor, enr, sut	*bla*_CMY-2_	*tet*B, *sul*I, *sul*II	+
30TE	tet, nal, sut	*bla*_CMY-2_	*tet*A, *tet*B, *sul*I, *sul*II	+
32.2 A	tet, nal, sut	*bla*_CMY-2_	*tet*A, *tet*B, *sul*I, *sul*II	+
33.3	tet, nit, nal, cip, enr	*bla*_CMY-2_	*tet*A, *tet*B, *sul*I, *sul*II	+
33.5	tet, nal, cip, nor, enr, sut	*bla*_CMY-2_	*tet*A, *tet*B, *sul*I, *sul*II	–
43.5	tet, gen, clo, nit, sut	*bla*_CMY-2_	*tet*A, *sul*I, *sul*II	–
44.2 A	tet, nal, sut	*bla*_CMY-2_	*tet*A, *sul*I	+
Human samples				
U79167	tet, nal, cip, nor, enr, sut	*bla*_CMY-2;_ *bla*_TEM-1_	*tet*A	+
U7895	tet, nal, cip, nor, enr, sut	*bla*_CMY-2_	*tet*A	–
T746	tet, nal, cip, nor, enr, sut	*bla*_CMY-2;_ *bla*_TEM-1_	*tet*B	–
U7772	tet, gen, nal,cip, nor, enr, sut	*bla*_CMY-2_	*tet*B	+
S8293	tet, nit, nal, nor, enr	*bla*_CMY-2_	*tet*B, *sul*II	+
S4474	nal, cip, nor, enr	*bla*_CMY-2_	–	+

tetracycline (tet), gentamicin (gen), chloramphenicol (clo), nitrofurantoin (nit), nalidixic acid (nal), ciprofloxacin (cip), norfloxacin (nor), enrofloxacin (enr), trimethoprim-sulfamethoxazole (sut)

(+) Presence

(−) Absence

ESBL production, by phenotypic testing, was not observed for any strain. In addition, the *bla*_TEM-1_ gene was found in 2 strains isolated from human.

To detect whether ISE*cp1* is upstream of *bla*_CMY-2_, PCR with a forward primer targeting the ISE*cp1* element and a reverse primer targeting the *bla*_CMY_ genes was performed, and the amplicons of positive strains were sequenced. Ten strains (6 from chicken carcasses and 4 from human infection) were positive, and sequencing confirmed that *bla*_CMY-2_ genes are linked to an upstream ISE*cp1*-like element.

By MLST, 11 sequence types (STs) were found. Three strains isolated from human infection (2 from urine and 1 from tissue) and one strain isolated from a chicken carcass belonged to ST354 (Fig. 1).

The AmpC-beta-lactamase-producers were classified within 13 PFGE types, showing high diversity among strains. Only two strains of ST354 from human urine and tissue (U79167 and T746 strains) showed 100% similarity (Fig. 1).

## Discussion

The emergence of third-generation cephalosporin-resistant Enterobacteriaceae, such as expressing ESBL and AmpC, in food-producing animals and their products has impacted the health of consumers, leading to the hypothesis that animals might become antimicrobial resistance sources and/or even contribute to the spread of these bacteria. Recent studies have shown that poultry and humans share similar antimicrobial resistance genes, and *E. coli* strain types, suggesting that transmission from poultry to humans may occur [2–4]. The presence of similar pAmpC in strains isolated from chicken meat and human clinical samples, in the same city and similar time period led us to compare the similarity of these strains by PFGE and MLST methodologies and to determine their antimicrobial resistance profiles to understand the dissemination of this mechanism of resistance.

In 2013, our group identified 8 pAmpC-producing *E. coli* isolates from chicken carcasses [12]. Near this time period, 6 pAmpC-producing *E. coli* were also isolated from patients with infection from a hospital in the same city where our study was conducted with chicken carcasses. All pAmpC of these strains, from chicken carcasses and human infection, were identified as the *bla*_CMY-2_ gene by sequencing. According to the literature, *bla*_CMY-2_ is the most common pAmpC gene identified from widespread human and veterinary medical cases [2–4]. Initially, in Brazil, pAmpC-producing bacteria were only observed in human medical settings. FOX-5 like and CMY-2-like were the first pAmpC beta-lactamases reported in Brazilian isolates and were detected in *E. coli* from patients in hospitals [10, 11]. Studies have shown an increase in the frequency of pAmpC in human clinical setting, but few studies have described the frequency of pAmpC in Enterobacteriaceae in Brazil [6, 11, 28, 29].

However, since 2015, studies have found pAmpC-producing bacteria in food-producing animals, mainly chicken meat in Brazil, leading to the hypothesis that this might become an infection source or reservoir that contributes to the spread of these bacteria. The *bla*_CMY-2_ gene is also the pAmpC variant more frequently found in food-producing animals in Brazil [12–16, 30]. Studies have detected a high prevalence of *bla*_CMY-2_ genes harbored on different plasmids in *E. coli* from poultry [13, 14]. In Brazil, da Silva and collaborators (2017) [16] found *bla*_CMY-2_ in avian pathogenic *E. coli* (APEC) from turkey, with airsacculitis, showing that this antimicrobial resistance mechanism can also be found in pathogenic strains.

The true rate of occurrence of pAmpC in strains of *E. coli* remains unknown because only a few surveillance studies have examined this resistance mechanism in Brazil [6]. Moreover, the lack of a standardized phenotypic method for the detection of AmpC-producing isolates contributes to underreporting in human clinical laboratories and veterinary medicine [5]. This higher frequency of pAmpC found in food-producing animals in recent years may be linked to changes in molecular epidemiology of AmpC beta-lactamase and to the indiscriminate use of antimicrobials in the production of these animals, which may be selecting this resistance mechanism.

Infections caused by pAmpC-producing bacteria limit therapeutic options since these organisms are usually resistant to all beta-lactam antibiotics, except cefepime, cefpirome, and the carbapenems [5]. In our study, all strains were considered multidrug-resistant and were nonsusceptible to at least 1 agent in 3 or more antimicrobial categories [27]. The highest frequencies of resistance to non-beta-lactam antimicrobials were for tetracycline (92.86%), nalidixic acid (92.86%) and sulfamethoxazole-trimethoprim (78.57%). In addition, by PCR, our strains showed that chicken meat is a reservoir of non-beta-lactam resistance genes such as *tet*A, *tet*B, *sul*I and *sul*II (Table 2), which corroborates the high frequency of phenotypic resistance for tetracycline and sulfamethoxazole-trimethoprim. In addition, two strains from humans have the *bla*_TEM-1_ gene, which encodes a beta-lactamase with a lower spectrum of action. The *bla*_pAmpC_ genes are usually present in mobile genetic elements, which carry resistance genes encoding other beta-lactamases and/or genes encoding resistance to other classes of antimicrobials, as pAmpC-producing bacteria are commonly multiresistant [5]. Co-resistance phenotypes are involved in the maintenance of resistance genes and plasmids in *E. coli* thus, the use of antimicrobials in animal production may also play a role in the selection of multidrug-resistant isolates in the animals’ environment [4].

A variety of genetic elements has been implicated in the mobilization of *bla*_ampC_ genes onto plasmids. It has been reported that beta-lactamase genes can be genetically linked to an upstream insertion elements, as ISE*cp1*. Many studies have shown that *bla*_AmpC_- type genes are associated with mobile genetic elements, including insertion sequences such as ISE*cp1*, most of which are carried on transferable plasmids [5, 31, 32]. In Brazil, only one study reported the presence of the ISE*cp* 1-*bla*_CMY-2_ gene present on a plasmid from an *E. coli* strain isolated from chickens [13]. In our study, six strains from chicken carcasses and four strains from humans infection (71.4% of the total strains) showed the *bla*_CMY-2_ gene linked to an upstream ISE*cp* 1-like element. This insertion element can be responsible for the transposition of *bla*_CMY-2_ to different plasmids and can also have an important role in the dissemination of CMY-2 beta-lactamases.

MLST is a methodology that can reflect the microevolution of the *E. coli* core genome, providing a true picture of the population structure of this bacterial species [4]. Eleven STs were found in this study (Fig. 1), all of which were reported both in birds and humans, with the exception of ST3177, which has never been reported in birds. All the STs have been reported in Brazil, with the exception of ST448 and ST3177 [4, 13, 33–42]. STs 10, 57, 93 and 117 were reported in avian pathogenic *E. coli* (APEC) and extraintestinal pathogenic *E. coli* (ExPEC) in Brazil, showing that these strains may be related to strains pathogenic, for both poultry and humans [38].

Four strains were grouped as ST354 (3 strains isolated from human infection and 1 strain isolated from a chicken carcass) suggested the possibility that they share the same clonal origin. However, PFGE revealed considerable heterogeneity among these strains. The most closely related strains were the 2 strains isolated from urine and tissue of human infection. PFGE also revealed 13 different PFGE types, with the dendrogram clearly showing a good distinction between the strains isolated from humans and chicken carcasses (Fig. 1). These data suggest a high diversity of strains that carry pAmpC genes and show possible parallel microevolution [4].

According to our study, we found a diverse *E. coli* population from both chicken carcasses and in human infection carrying the *bla*_CMY-2_ gene. Some studies also concluded that dissemination of AmpC-producing *E. coli* does not occur by clonal strains in these hosts [43, 44]. However, in our study, the presence of ISE*cp1* upstream of *bla*_*C*MY-2_ in some strains suggests that mobile genetic elements are being disseminated between bacteria from humans and animals, mainly poultry.

Antimicrobials are normally used in animal husbandry as veterinary drugs or feed additives [45]. Although a withdrawal time for antimicrobial use is required before the animal is sacrificed for sale, Wang and collaborators (2017) found residues of antimicrobials in meat and even detected some human antimicrobials, that are not used as veterinary drugs. The spread of antimicrobial resistance genes in poultry may be associated with the prophylactic use of cephalosporins injected into eggs to control *E. coli* omphalitis in broiler chickens [46]. In Brazil, third-generation cephalosporins have been associated with *in ovo* vaccination on the 18th day of incubation because the vaccine can also select antimicrobial resistant bacteria in poultry [33].

Further research about the dissemination of resistant bacteria need to be conducted in a given time and geographical area to trace the flow of resistant bacteria because there are few studies about this dissemination [4]. Our study allows us to understand some aspects of the dissemination of this resistance mechanism in a restricted area, which is important step for developing strategies aimed at preventing the propagation of this resistance through food ingestion. These data show the presence of the *bla*_CMY-2_ gene linked with an ISE*cp1*-type insertion element in both chicken carcasses and in human infection in a restricted region. Our results suggest the presence of highly diverse strains that harbor pAmpC, indicating no clonal dissemination. In a “One-Health” context, continuous collaboration among professionals in human and animal healthcaree, the food industry and the environmental sector is needed to characterize the occurrence and routes of dissemination of these antimicrobial resistance determinants.

## Conclusion

Since Brazil is one of the largest exporters of chicken meat in the world, surveillance studies are essential to identify resistance genes and bacterial clones that may spread from chickens to humans. Our results show the presence of highly diverse strains that harbor pAmpC, indicating no clonal dissemination. However, the presence of *bla*_CMY-2_, linked to the ISE*cp1* element, was present both in chicken meat and human infection, suggesting that mobile genetic elements can be responsible for the spread of this resistance mechanism in this restricted area. Therefore, continuous monitoring and comparative analyses of resistant bacteria from humans and food-producing animals are needed.

## Data Availability

All the data supporting our findings are contained in the manuscript. The raw data and scientific records are saved in our laboratory and can be obtained from the corresponding author per a reasonable request.

## Abbreviations

APEC: Avian pathogenic *E. coli*
CLSI: Clinical and Laboratory Standards Institute
Embrapa: Brazilian Agricultural Research Corporation
ESBL: Extended-spectrum beta-lactamase
ExPEC: Extraintestinal pathogenic *E. coli*
MLST: Multilocus sequence typing
pAmpC: Plasmid-mediated AmpC
PCR: Polymerase chain reaction
PFGE: Pulsed-field gel electrophoresis
PMQR: Plasmid mediated quinolone resistance
ST: Sequence typing
UEL: State University of Londrina
UNICAMP: State University of Campinas

## References

[1] Cyoia PS, Koga VL, Nishio EK, Houle S, Dozois CM, de Brito KCT, de Brito BG, Nakazato G, Kobayashi RKT (2019). Distribution of ExPEC virulence factors, *bla*_CTX-M_, *fos*A3, and *mcr*-1 in *Escherichia coli* isolated from commercialized chicken carcasses. Front Microbiol.

[2] Lazarus B, Paterson DL, Mollinger JL, Rogers BA (2015). Do human extraintestinal *Escherichia coli* infections resistant to expanded-spectrum cephalosporins originate from food-producing animals? A systematic review. Clin Infect Dis.

[3] Liebana E, Carattoli A, Coque TM, Hasman H, Magiorakos AP, Mevius D, Peixe L, Poirel L, Schuepbach-Regula G, Torneke K, Torren-Edo J, Torres C, Threlfall J (2013). Public health risks of enterobacterial isolates producing extended-spectrum β-lactamases or AmpC β-lactamases in food and food-producing animals: an EU perspective of epidemiology, analytical methods, risk factors, and control options. Clin Infect Dis.

[4] Ewers C, Bethe A, Semmler T, Guenther S, Wieler LH (2012). Extended-spectrum β-lactamase-producing and AmpC-producing *Escherichia coli* from livestock and companion animals, and their putative impact on public health: a global perspective. Clin Microbiol Infect.

[5] Jacoby GA (2009). AmpC β-lactamases. Clin Microbiol Rev.

[6] Da Silva Dias RC, Borges-Neto AA, D’Almeida Ferraiuoli GL, de Oliveira MP, Riley LW, Moreira BM (2008). Prevalence of AmpC and other β-lactamases in enterobacteria at a large urban university hospital in Brazil. Diagn Microbiol Infect Dis.

[7] Pérez-Pérez FJ, Hanson ND (2002). Detection of plasmid-mediated AmpC β-lactamases genes in clinical isolates by using multiplex PCR. J Clin Microbiol.

[8] Rensing KL, Abdallah HM, Koek A, Elmowalid GA, Vandenbroucke-Grauls CMJE, Al Naiemi N, van Dijk K (2019). Prevalence of plasmid-mediated AmpC in Enterobacteriaceae isolated from humans and from retail meat in Zagazig. Egypt Antimicrob Resist Infect Control.

[9] Meini Simone, Tascini Carlo, Cei Marco, Sozio Emanuela, Rossolini Gian Maria (2019). AmpC β-lactamase-producing Enterobacterales: what a clinician should know. Infection.

[10] Castanheira M, Pereira AS, Nicoletti AG, Pignatari ACC, Barth AL, Gales AC (2007). First report of plasmid-mediated *qnr*A1 in a ciprofloxacin-resistant *Escherichia coli* strain in Latin America. Antimicrob Agents Chemother.

[11] Pavez M, Neves P, Dropa M, Matté MH, Grinbaum RS (2008). Elmor de Araújo MR, Mamizuka EM, Lincopan N. emergence of carbapenem-resistant *Escherichia coli* producing CMY-2-type AmpC β-lactamase in Brazil. J Med Microbiol.

[12] Koga VL, Rodrigues GR, Scandorieiro S, Vespero EC, Oba A, de Brito BG, de Brito KCT, Nakazato G, Kobayashi RKT (2015). Evaluation of the antibiotic resistance and virulence of *Escherichia coli* strains isolated from chicken carcasses in 2007 and 2013 from Paraná. Brazil Foodborne Pathog Dis.

[13] Casella T, Haenni M, Madela NK, de Andrade LK, Pradela LK, de Andrade LN, Darini ANC, Madec J-Y, Nogueira MCL (2018). Extended-spectrum cephalosporin-resistant *Escherichia coli* isolated from chickens and chicken meat in Brazil is associated with rare and complex resistance plasmids and pandemic ST lineages. J Antimicrob Chemother.

[14] Ferreira JC, Penha Filho RAC, Andrade LN, Berchieri Junior A, Darini ALC (2017). Diversity of plasmids harbouring *bla*_CMY2_ in multidrug-resistant *Escherichia coli* isolated from poultry in Brazil. Diagn Microbiol Infect.

[15] Botelho LAB, Kraychete GB (2015). Costa e Silva JL, Regis DVV, Picão RC, Moreira BM, Bonelli RR. Widespread distribution of CTX-M and plasmid-mediated AmpC β-lactamases in *Escherichia coli* from Brazilian chicken meat. Mem Inst Oswaldo Cruz.

[16] Da Silva KC, Cunha MP, Cerdeira L, de Oliveira MG, de Oliveira MC, Gomes CR, Lincopan N, Knöbl T, Moreno AM (2017). High-virulence CMY-2- and CTX-M2-producing avian pathogenic *Escherichia coli* strains isolated from commercial turkeys. Diagn Microbiol Infect Dis.

[17] Woodford N, Fagan EJ, Ellington MJ (2005). Multiplex PCR for rapid detection of genes encoding CTX-M extended-spectrum β-lactamases. J Antimicrob Chemother.

[18] Dallenne C, da Costa A, Decré D, Favier C, Arlet G (2010). Development of a set of multiplex PCR assays for the detection of genes encoding important β-lactamases in Enterobacteriaceae. J Antimicrob Chemother.

[19] Jouini A, Vinué L, Slama KB, Sáenz Y, Klibi N, Hammami S, Boudabous A, Torres C (2007). Characterization of CTX-M and SHV extended-spectrum β-lactamases and associated resistance genes in *Escherichia coli* strains of food samples in Tunisia. J Antimicrob Chemother.

[20] Dierikx C, van Essen-Zandbergen A, Veldman K, Smith H, Mevius D (2010). Increased detection of extended spectrum beta-lactamase producing *Salmonella enterica* and *Escherichia coli* isolates from poultry. Vet Microbiol.

[21] Cattoir V, Poirel L, Rotimi V, Soussy C-J, Nordmann P (2007). Multiplex PCR for detection of plasmid-mediated quinolone resistance *qnr* genes in ESBL-producing enterobacterial isolates. J Antimicrob Chemother.

[22] Li Q, Sherwood JS, Logue CM (2007). Characterization of antimicrobial resistant *Escherichia coli* isolated from processed bison carcasses. J Appl Microbiol.

[23] Liao W, Jiang J, Xu Y, Yi J, Chen T, Su X, Pan S, Wei X, Li Y (2010). Survey for β-lactamase among bacterial isolates from Guangzhou, China hospitals between 2005–2006. J Antibiot (Tokyo).

[24] Clinical Laboratory Standards Institute (2008). Performance standards for antimicrobial disk and dilution susceptibility test for bacteria isolated from animals; 3^rd^ edition, CLSI document M31-A3.

[25] Clinical Laboratory Standards Institute (2013). Performance standards for antimicrobial susceptibility testing; 23th informational supplement M100-S23.

[26] Jarlier V, Nicolas M-H, Fournier G, Philippon A (1988). Extended broad-spectrum beta-lactamases conferring transferable resistance to newer beta-lactam agents in Enterobacteriaceae: hospital prevalence and susceptibility patterns. Rev Infect Dis.

[27] Magiorakos AP, Srinivasan A, Carey RB, Carmeli Y, Falagas ME, Giske CG, Harbarth S, Hindler JF, Kahlmeter G, Olsson-Liljequist B, Paterson DL, Rice LB, Stelling J, Struelens MJ, Vatopoulos A, Weber JT, Monnet DL (2012). Multidrug-resistant, extensively drug-resistant and pandrug-resistant bacteria: an international expert proposal for interim standard definitions for acquired resistance. Clin Microbiol Infect.

[28] Rocha DA, Campos JC, Passadore LF, Sampaio SC, Nicodemo AC, Sampaio JL (2016). Frequency of plasmid-mediated AmpC β-lactamases in *Escherichia coli* isolates from urine samples in São Paulo. Brazil Microb Drug Resist.

[29] Campana EH, Barbosa PP, Fehlberg LCC, Gales AC (2013). Frequency of plasmid-mediated AmpC in Enterobacteriaceae isolated in a Brazilian teaching hospital. Braz J Microbiol.

[30] Moura Q, Fernandes MR, Silva KC, Montes DF, Esposito F, Dropa M, Noronha C, Moreno AM, Landgraf M, Negrão FJ, Lincopan N (2018). Virulent nontyphoidal *Salmonella* producing CTX-M and CMY-2 β-lactamases from livestock, food and human infection. Brazil Virulence.

[31] Guo Yu-Fang, Zhang Wen-Hui, Ren Si-Qi, Yang Lin, Lü Dian-Hong, Zeng Zhen-Ling, Liu Ya-Hong, Jiang Hong-Xia (2014). IncA/C Plasmid-Mediated Spread of CMY-2 in Multidrug-Resistant Escherichia coli from Food Animals in China. PLoS ONE.

[32] Fernández-Alarcón Claudia, Singer Randall S., Johnson Timothy J. (2011). Comparative Genomics of Multidrug Resistance-Encoding IncA/C Plasmids from Commensal and Pathogenic Escherichia coli from Multiple Animal Sources. PLoS ONE.

[33] Ferreira JC, Penha Filho RAC, Kuaye APY, Andrade LN, Chang YF, Darini ALC (2018). Virulence potential of commensal multidrug resistant *Escherichia coli* isolated from poultry in Brazil. Infect Genet Evol.

[34] Sellera FP, Fernandes MR, Moura Q, Carvalho MPN, Lincopan N (2018). Extended-spectrum-β-lactamase (CTX-M)-producing *Escherichia coli* in wild fishes from a polluted area in the Atlantic Coast of South America. Mar Pollut Bull.

[35] Pietsch M, Eller C, Wendt C, Holfelder M, Falgenhauer L, Fruth A, Grössl T, Leistner R, Valenza G, Werner G, Pfeifer Y, RESET Study Group (2017). Molecular characterization of extended-spectrum β-lactamase (ESBL)-producing *Escherichia coli* isolates from hospital and ambulatory patients in Germany. Vet Microbiol.

[36] de Souza da-Silva AP, de Sousa VS, Martins N, da Silva Dias RC, Bonelli RR, Riley LW, Moreira BM (2017). *Escherichia coli* sequence type 73 as a cause of community acquired urinary tract infection in men and women in Rio de Janeiro. Brazil Diagn Microbiol Infect Dis.

[37] Müller A, Stephan R, Nüesch-Inderbinen M (2016). Distribution of virulence factors in ESBL-producing *Escherichia coli* isolated from the environment, livestock, food and humans. Sci Total Environ.

[38] Maluta Renato Pariz, Logue Catherine Mary, Casas Monique Ribeiro Tiba, Meng Ting, Guastalli Elisabete Aparecida Lopes, Rojas Thaís Cabrera Galvão, Montelli Augusto Cezar, Sadatsune Teruê, de Carvalho Ramos Marcelo, Nolan Lisa Kay, da Silveira Wanderley Dias (2014). Overlapped Sequence Types (STs) and Serogroups of Avian Pathogenic (APEC) and Human Extra-Intestinal Pathogenic (ExPEC) Escherichia coli Isolated in Brazil. PLoS ONE.

[39] Ben Sallem R, Ben Slama K, Rojo-Bezares B, Porres-Osante N, Jouini A, Klibi N, Boudabous A, Sáenz Y, Torres C (2014). InCI1 plasmids carrying *bla*_CTX-M-1_ or *bla*_CMY-2_ genes in *Escherichia coli* from healthy humans and animals in Tunisia. Microb Drug Resist.

[40] Voets GM, Fluit AC, Scharringa J, Schapendonk C, van den Munckhof T, Leverstein-van Hall MA, Stuart JC (2013). Identical plasmid AmpC beta-lactamase genes and plasmid types in *E. coli* isolates from patients and poultry meat in the Netherlands. Int J Food Microbiol.

[41] Pitondo-Silva A, Minarini LA, Camargo IL, Darini AL (2009). Clonal relationship determined by multilocus sequence typing among enteropathogenic *Escherichia coli* isolated in Brazil. Can J Microbiol.

[42] Hasan B, Sandegren L, Melhus A, Drobni M, Hernandez J, Waldenström J, Alam M, Olsen B (2012). Antimicrobial drug-resistant *Escherichia coli* in wild birds and free-range poultry. Bangladesh Emerg Infect Dis.

[43] De Been M, Lanza VF, de Toro M, Scharringa J, Dohmen W, Du Y, Hu J, Lei Y, Li N, Tooming-Klunderud A, Heederik DJ, Fluit AC, Bonten MJ, Willems RJ, de la Cruz F, van Schaik W (2014). Dissemination of cephalosporin resistance genes between *Escherichia coli* strains from farm animals and humans by specific plasmid lineages. PLoS Genet.

[44] Börjesson S, Ny S, Egervän M, Bergström J, Rosengren A, Englund S, Löfmark S, Byfords S (2016). Limited dissemination of extended-spectrum β-lactamase- and plasmid-encoded AmpC-producing *Escherichia coli* from food and farm animals. Sweden Emerg Infect Dis.

[45] Wang H, Ren L, Yu X, Hu J, Chen Y, He G, Jiang Q (2017). Antibiotic residues in meat, milk and aquatic products in Shanghai and human exposure assessment. Food Control.

[46] Dutil L, Irwin R, Finley R, Ng LK, Avery B, Boerlin P, Bourgault AM, Cole L, Daignault D, Desruisseau A, Demczuk W, Hoang L, Horsman GB, Ismail J, Jamieson F, Maki A, Pacagnella A, Pillai DR (2010). Ceftiofur resistance in *Salmonella enterica* serovar Heidelberg from chicken meat and humans. Canada Emerg Infect Dis.

